# Laparoscopic adrenal surgery in children: Lessons from a single centre experience

**DOI:** 10.4103/0972-9941.78346

**Published:** 2011

**Authors:** Sudhir Sukumar, Santosh Jadhav, Balagopal Nair, Sanjay H Bhat, Ginil P Kumar, Georgie Mathew

**Affiliations:** Department of Urology, Amrita Institute of Medical Sciences and Research Centre, Kochi, India

**Keywords:** Adrenal gland, child, laparoscopy

## Abstract

**PURPOSE:**

Although commonly performed in adults, laparoscopic adrenalectomy in children is performed only in centres with advanced laparoscopic expertise.

**MATERIALS AND METHODS:**

This is a retrospective analysis of laparoscopic adrenalectomies performed at a single centre between January 2003 and May 2010. After preoperative evaluation with biochemical assays and radiologic imaging, surgery was performed by using the lateral transabdominal approach in all patients.

**RESULTS:**

Ten laparoscopic adrenalectomies (including three bilateral) were performed in seven children, with a mean age of 9.6 years. The tumours ranged from 2 – 7 cms in size. The operative durations were 75 – 130 minutes (unilateral) and 250 – 270 minutes (bilateral). Operative blood loss was minimal. There were no open conversions, but terminal hand assistance was required in one large right pheochromocytoma. The postoperative hospital stay ranged from 3 – 10 days. The final pathological diagnoses included pheochromocytoma, hyperplasia and neuroblastoma. Follow-up at 24 – 87 months was uneventful.

**CONCLUSION:**

With adequate experience in laparoscopy, it is possible to perform adrenalectomy in selected children.

## INTRODUCTION

The laparoscopic approach for excision of the adrenal gland was first described by Gagner *et al*. in 1992.[1] Over the years, several series have established laparoscopic adrenalectomy (LA) as the gold standard for the removal of adrenal lesions of almost any pathology in adults.[2–4] Utilization of laparoscopy for adrenalectomy in the pediatric and adolescent age groups is much lower, due to the relative infrequency of adrenal masses in this patient population associated with a relatively higher incidence of malignancy;[5] other factors like small body size and inexperience with smaller laparoscopic instrumentation may also limit the enthusiasm among surgeons for this approach.[6]

This study reviews our experience with LA in children, at a single referral centre.

## MATERIALS AND METHODS

This is a retrospective study of all laparoscopic adrenal surgeries performed on pediatric patients at our institute from January 2003 to May 2010. Patient medical records were reviewed for demographic data, preoperative evaluation and diagnosis, as also preoperative details, postoperative hospital stay, complications, histopathological features and follow-up data.

All patients underwent preoperative biochemical evaluation to rule out functional tumours. Radiological imaging with contrast-enhanced computed tomography (CT) or magnetic resonance imaging (MRI) scans was done in all patients to assess the size, side, local extent and distant metastasis. Additionally, metaiodobenzylguanidine scans were carried out during evaluation of functioning adrenal medullary lesions.

As in adults, the blood pressures of children with biochemical or clinical evidence of phaeochromocytoma were controlled prior to surgery with alpha blockade, using doxazocin and beta blockade when necessary.

### Surgical Technique

**Figure 1:**
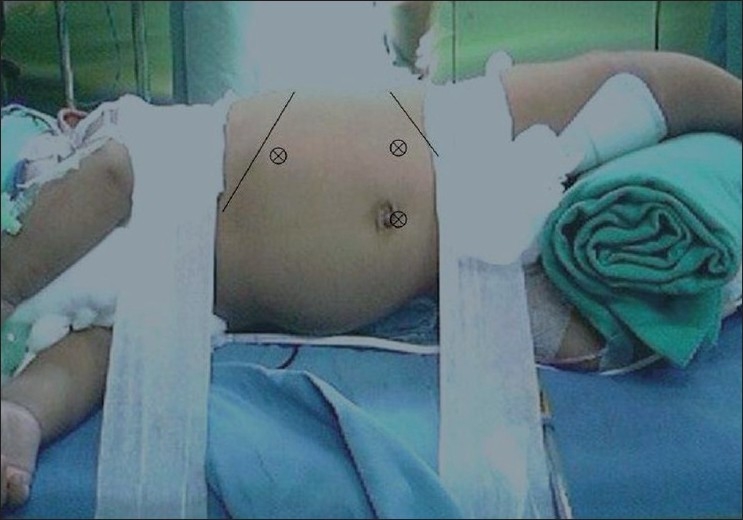
Port positions for left laparoscopic adrenalectomy in a child

All cases were performed by the standard lateral transperitoneal approach, which has been extensively described. The patients were positioned in the 45° lateral decubitus position and a sub-umbilical 5 mm or 10 mm port was used for a 30° telescope. Two 3 mm or 5 mm working ports were used — one in the iliac region and the other subcostal port in the midclavicular line [Figure 1]; an additional 3 mm epigastric port was used for liver retraction in right-sided procedures. Carbon dioxide was used for insufflation maintaining pneumoperitoneum at a pressure of 8 – 10 mm Hg. When bilateral LA was performed in a single stage, the patients were re-positioned and re-draped after completion on one side. For the left-sided procedures, early control of the adrenal vein was attained at its origin from the renal vein. This was accomplished after colonic and splenopancreatic mobilisation, without direct handling of the adrenal gland itself. For right-sided procedures, the right lobe of the liver was mobilised and retracted upwards after dividing the right coronary ligament. The sub-hepatic inferior vena cava was exposed to approach the adrenal vein at the superomedial corner of the adrenal gland. After division of the adrenal veins, the gland was dissected free. The specimens were routinely placed in indigenously prepared plastic bags and retrieved through the umbilical port, extending it judiciously when necessary.

## RESULTS

Ten adrenalectomies were performed in seven children during the seven-year period of study. The four boys and three girls had a mean age of 9.6 years and a mean body-weight of 28 kgs. All the patients were symptomatic on presentation [Table 1] and none had antenatally detected masses or incidentalomas. Preoperative diagnosis were phaeochromocytoma (n = 4), Cushing’s disease refractory to pituitary surgery (n = 2) and ganglioneuroma (n = 1). Three children underwent bilateral single-stage adrenalectomy, while four underwent unilateral adrenalectomy — three on the left side and one on the right side.

**Table 1:** Demographics, perioperative data and final histopathology of seven patients

Sex / Age (Years)	Body Weight (kgs)	Presentation	Side / Size (cm)	Operative time (min)	Postoperative Stay (days)	Pathology
M /16	39	Hypertension	L / 5	75	4	Normal Adrenal
F / 12	55	Cushing’s syndrome	R / 5	270	5	Cushing’s disease [Adrenal Hyperplasia]
			L / 5		
F / 14	34	Hypertension	R / 5	130	4	Pheochromocytoma
F / 1	8	WDHA	L / 3.7	120	3	Ganglioneuroma
M / 14	25	Hypertension	R / 7	250	6	Pheochromocytoma
			L / 5		
M / 10	32	Cushing’s syndrome	R / 5	270	5	Cushing’s disease [Adrenal Hyperplasia]
			L / 5		
M / 0.5	3	Hypertension	L / 2.3	120	10	Neuroblastoma

WDHA – Watery Diarrhoea-Hypokalaemia-Alkalosis Syndrome

On radiological imaging, the sizes of the adrenal masses ranged from 2 to 7 cm (mean 4.8 cm) in the longest dimension. The mean operative time was 111 minutes for unilateral and 263 minutes for bilateral cases. Estimated blood loss was negligible (< 50 ml) in all cases. The same surgical technique was followed in all patients and early adrenal vein ligation was successfully accomplished in all 10 procedures. Two patients with phaeochromocytoma developed intraoperative hemodynamic fluctuations in spite of adequate preoperative preparation with alpha blockade and intravenous hydration. There were no open conversions, but terminal hand assistance was employed in one child to dissect and retrieve a 7cm right phaeochromocytoma with retrocaval extension.

Postoperative hospital stay averaged 5.3 days. One infant developed postoperative septic arthritis of the hip joint that required prolonged hospital stay and parenteral antibiotics. Those with Cushing’s disease were transferred to the endocrinology unit for further management. There was no mortality in this series of seven patients.

The final histopathology [Table 1] revealed a normal adrenal gland in a teenage boy with uncontrolled hypertension, which required three anti-hypertensive drugs. His urinary vanillylmandelic acid levels were elevated and the preoperative CT scan was initially interpreted as a left adrenal mass; in retrospect, it was probably just a very prominent splenic notch. The histology of the female infant who presented with features of the watery diarrhoea-hypokalaemia-alkalosis syndrome confirmed ganglioneuroma. The male infant who was evaluated for hypertension was operated with a suspicion of phaeochromocytoma, but the final histopathology was suggestive of a localised neuroblastoma.

The follow-up duration ranges from 24 to 87 months (mean — 54.4 months). The child with stage I neuroblastoma is on follow-up and continues to be symptom-free at 24 months. The children who had bilateral adrenalectomy are on replacement therapy with hydrocortisone and fludrocortisone. All the children have had a resolution of the clinical and biochemical markers of adrenal gland hyperfunction. None of the children have shown clinical or radiological signs of local recurrence or distant metastases during the follow-up.

## DISCUSSION

The adrenal gland is considered suitable for laparoscopic resection because of its small size and retroperitoneal location.[7] It has established advantages over the open procedure in adults; these advantages are now being extrapolated to the pediatric age group also. Indications for adrenal surgery differ in adults and children. Literature reveals neuroblastic tumours to be the most common adrenal lesions, requiring excision in the pediatric age group;[6,8] there was one neuroblastoma and one ganglioneuroma in our single centre experience. The safety and feasibility of LA for all stages of neuroblastomas in pediatric patients (including infants) has been previously documented.[6–9] In fact, LA has even been suggested as an option for residual tumours that have a favourable cytoreductive response to chemotherapy.[10,11]

Unlike in adults, suspected phaeochromocytomas in children and adolescents need to be thoroughly evaluated to rule out bilateral lesions, extra-adrenal lesions as well as syndromic associations like multiple endocrine neoplasia, von Recklinghausen disease, tuberous sclerosis, Sturge-Weber syndrome and von Hippel Lindau syndrome.[6,7] As in adults, children also require preoperative preparation, with appropriate alpha adrenergic blockers. Operative precautions like early control of adrenal veins and minimal handling of the gland are followed, as for adults. The intraoperative haemodynamic fluctuations that may occur in patients with phaeochromocytomas are thought to be less common in laparoscopy than in open surgery and can usually be controlled with temporary cessation of the procedure and appropriate medical measures.[12]

Two children with Cushing’s disease and one with phaeochromocytomas underwent bilateral LA under the same anaesthesia. The boy with bilateral phaeochromocytomas, as part of the von Hippel Lindau Syndrome, had a small left adrenal mass that was first excised; the larger right adrenal mass had significant retrocaval extension that necessitated insertion of the surgeon’s non-dominant hand for the final stages of dissection. No special hand-assist ports were used and excision was completed with laparoscopic instrumentation. As the same incision was then used for retrieval of the specimen, the cosmesis was also not altered. The terminal hand assist is a modification we had previously used successfully in adults to complete difficult laparoscopic procedures, without an open conversion.[13] It facilitated overcoming difficult mobilisation in the later stages of surgery, while avoiding a hand port early on in the dissection. In this child, it was useful in excising the relatively large mass from the comparatively smaller intraperitoneal space.

Bilateral LA in children is not frequently reported and can be performed under one anaesthesia[11,14] or as a staged procedure.[6] Ideally partial adrenal gland preservation should be attempted in children undergoing bilateral adrenalectomy;[15] this was accomplished in the boy with the von Hippel Lindau syndrome, where a portion of normal appearing tissue from the right adrenal gland was left behind after excising the large tumour. The use of intraoperative ultrasound through laparoscopic ports has been prescribed to accurately delineate the adenoma and preserve the normal cortex,[16] but this is presently not available at our centre. In the children with refractory Cushing’s disease, the need for complete ablation precluded conservative surgery.

Although other series has successfully used the retroperitonescopic approach for pediatric LA,[17–19] we routinely prefer the transperitoneal approach in pediatric laparoscopy. Of late we have started employing the retroperitoneoscopic approach for adrenalectomy in selected adults and as our experience improves, we hope to attempt this in children as well.

## CONCLUSION

LA can be considered to be equally safe and effective in pediatric patients as in adults and is applicable for most pathologies. However, patient selection for laparoscopy is crucial and is dependant on the child’s body habitus, as well as the experience of the surgical and anaesthetic teams. A low threshold for open conversion in the early phase of the learning curve is recommended. Terminal hand assist may be beneficial in difficult cases.
